# Dental healthcare utilization and mortality: a population-based prospective cohort study in southern Sweden

**DOI:** 10.2340/aos.v85.46190

**Published:** 2026-05-29

**Authors:** Martin Lindström, Aron Naimi-Akbar, Emma Lindström, Maria Rosvall, Mirnabi Pirnouzifard

**Affiliations:** a Social Medicine and Health Policy, Department of Clinical Sciences and Centre for Primary Health Care Research, Lund University, Malmö, Sweden; b Health Technology Assessment-Odontology (HTA-O), Faculty of Odontology, Malmö University, Malmö, Sweden; c Department of Speech Language Pathology, Faculty of Arts, Theology and Psychology, Åbo Akademi University, Turku, Finland; d Department of Community Medicine and Public Health, Sahlgrenska Academy, Institute of Medicine, University of Gothenburg, Gothenburg, Sweden

**Keywords:** dental healthcare utilization, oral health, mortality, Sweden

## Abstract

**Objective:**

The aim was to investigate associations between dental healthcare utilization and all-cause, cardiovascular (CVD), cancer, and other-cause mortality.

**Materials and methods:**

A postal public health survey was distributed in the autumn of 2008 to a stratified random sample of the population aged 18–80 in Scania, southern Sweden (response rate 54.1%). Baseline survey data were linked to mortality data, creating a prospective cohort study with 8.3-year follow-up. The present study entails 26,115 respondents. Associations between dental healthcare utilization and mortality were investigated in survival analyses and analyses with a nested case–control design.

**Results:**

In all, 68.4% had visited dental healthcare <1 year ago; 19.0%, 1–2 years ago; 6.7%, 3–5 years ago; 5.5%, >5 years ago; and 0.4%, never. The groups that had visited dental healthcare 1–2 years ago, 3–5 years ago, and >5 years ago displayed increased hazard ratios of all-cause mortality throughout the multiple- adjusted survival analyses compared to the <1-year reference group. Conditional logistic regression analyses with a nested case–control design confirmed results with a clearer gradient in all-cause mortality. CVD, cancer, and other-cause mortality showed some significant associations with dental healthcare utilization.

**Conclusions:**

Dental healthcare utilization was associated with all-cause mortality, with a clear gradient in the nested case–control design.

## Introduction

There is a connection between oral health problems and mortality. A number of studies have reported a positive association between oral health problems and mortality, including all-cause, cardiovascular diseases (CVD) (ischemic heart disease [IHD] and stroke), respiratory, and cancer mortality [1–4]. Multiple oral health problems are associated with higher mortality [5]. However, no previous studies have studied associations between dental healthcare utilization and mortality.

Oral health includes conditions such as dental caries, dental plaque, dental calculus, gingival inflammation, periodontitis, tooth loss, missing teeth, and a range of masticatory dysfunctions [2]. Dental plaque bacteria can invade the blood vessels of the gingiva, which may result in bacteremia [6]. Tooth loss and, to a lesser extent, self-reported health of teeth and gums are markers for IHD, peripheral vascular disease (PVD), and all-cause mortality. Tooth loss is also a risk marker for heart failure [7]. Periodontitis is a potential pathway to persistent low-grade systemic inflammation [8, 9], which, according to the American College of Cardiology/American Heart Association (ACC/AHA), is an important risk marker for CVDs. The ACC/AHA has included the inflammation marker C-reactive protein (CRP) as an established risk marker in clinical risk assessment of CVDs in the USA [10]. Periodontitis may contribute to these inflammatory processes, which may accelerate atherosclerosis and lead to coronary heart disease [11, 12]. Tooth loss may affect the ability to eat and may thus have an impact on the intake of key nutrients linked to CVD and cancer mortality [13]. Periodontitis and subsequent tooth loss may also be indirectly connected with higher mortality because it causes poor nutrition and poor eating behaviors resulting in malnutrition, diabetes, and disability [14, 15]. Cognitive status and sensorimotor control may impact oral health due to food retention and impaired hygiene, for example, forgetting to brush teeth. Older adults also have a higher risk of xerostomia. Poor oral health is also a strong risk factor for aspiration pneumonia, which may lead to death, particularly in frail older people [16].

The association between oral health and mortality has been examined based on assessments of oral health by clinical examination [17–20]. Other studies have shown that both objective and subjective measures of oral health are risk factors significantly associated with mortality [21]. In contrast, studies investigating associations between frequency of visits to dental healthcare**,** dental healthcare utilization, and mortality are very scarce or nonexistent. The World Health Organization recommends that all countries adopt specific strategies for the improvement of oral health, particularly for the elderly. Such strategies include improved dental healthcare, increased dental healthcare utilization, and recruitment of dental healthcare professionals [22]. The connections between oral health and mortality thus call for studies of associations between dental healthcare utilization and mortality.

**Figure 1:**
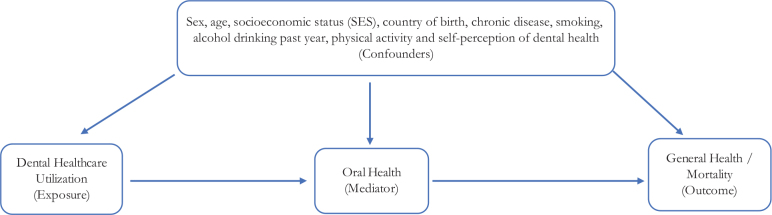
Model of associations between dental healthcare utilization, oral health, and general health/mortality. The steps dental healthcare utilization (exposure), oral health (mediator), and general health/mortality (outcome) are affected by demographic, socioeconomic, chronic disease, health-related behaviors, and self-perceived dental health factors (confounders).

There are some *a priori* preconditions in Sweden for socioeconomic oral health inequalities because the public subsidies for dental healthcare are less generous than the general health insurance. Social inequalities in healthcare utilization have also been observed in neighboring countries [23]. The current Swedish system started earlier than the study period of this study. Sweden has a general dental healthcare support (*Allmänt Tandvårdsbidrag/ATB*) to financially support and encourage regular dental and oral health examinations. This general dental healthcare support comprises free dental care until the age of 19, support of 300 SEK/year to individuals in the age interval 30–64, and 600 SEK/year in the age intervals 24–29 and 65 and above. Special dental healthcare support (*Särskilt Tandvårdsbidrag/STB*) may also be granted to particular groups with certain diagnoses and functional disabilities. A general high-cost protection (*Högkostnadsskydd*) provides financial aid to individuals with high dental healthcare needs. In the cost interval 3000–15,000 SEK, the high-cost protection is 50% of the reference price. If the cost exceeds 15,000 SEK, the high-cost protection is 85% of the reference price. From 1 January 2026, 90% of active treatments are paid for by public insurance for patients 67 years old and above. Basic dental health examinations are not included in this insurance. The present study exclusively examines a time period prior to this reform. In sum, this system of cost subsidies encourages the utilization of dental healthcare in the population, although it is not as generous as the health insurance in the general healthcare system in Sweden. The associations between dental healthcare utilization and mortality have, to our knowledge, not been investigated in prospective cohort analyses. Dental healthcare utilization of the dental healthcare system affects oral health, and oral health affects mortality as well as other health outcomes. Dental healthcare utilization, oral health, and mortality in this hypothesized chain of causality are all associated with and affected by demographic, socioeconomic, chronic disease, lifestyle factors, and self-perception of dental health (Figure 1).

The aim of this study is to investigate associations between dental healthcare utilization and all-cause, CVD, cancer, and other-cause mortality in a study with a prospective cohort design, including analyses with a nested case–control design.

## Methods

### Study design

This was a prospective cohort study based on data from a public health survey with linkage to the National Cause of Death Register through the Swedish 10-digit Personal Identity Number [24]. The data were also analyzed with a nested case–control study design.

### Study population

A public health survey was conducted in Scania, the southernmost part of Sweden, in the autumn of 2008. The cross- sectional survey population was sampled with a stratified sample of the register population aged 18–80 years. A postal invitation letter including a questionnaire was posted to the sample population. The invitation letter was followed by three postal letters of reminder. The questionnaire could also be completed online. The number of final respondents is 28,198, with a 54.1% response rate. The cross-sectional baseline questionnaire survey was performed by *Region Skåne* (Scania), which is the regional public authority responsible for the regional healthcare system. The survey questionnaire entails 134 items on demographic, socioeconomic, self-reported general and psychological health (e.g. GHQ12, SF36), social capital, social support, work conditions, health-related behaviors, and items related to security. Geographical stratification was based on age, sex, and education to obtain statistical power in all approximately 60 geographical areas (municipalities and parts of major cities). The sample was selected from the public national population register administered by *Statistics Sweden*. *Statistics Sweden* also created the population weight. The population weight compensates for stratification to reach representativeness with regard to the parameter population in the corresponding age interval. The cross-sectional survey (baseline) data were linked to register-based causes of death mortality data (*Dödsorsaksregistret*) from the National Board of Health and Welfare (*Socialstyrelse*n), creating a closed prospective cohort population.

The study was approved by the Ethics Committee (*Etikprövningsnämnden*) in Lund (No. 2010/343).

### Dependent variable

All-cause and diagnosis-specific mortality was followed prospectively from 27 August to 14 November 2008 (date depends on registration date of respondents’ individual answers) until the end of follow-up on 31 December 2016 (8.3 years onwards), or until death. Causes of death were defined according to International Classification of Diseases 10 (ICD 10). The 10-digit personal identity number system in Sweden makes the linkage of baseline survey questionnaire data with causes of death data from the Swedish *National Board on Health and Welfare* (*Socialstyrelsen*) possible. The linkage was performed by a third party (private company). The 10-digit personal identity numbers were deleted from the dataset before delivery to the researchers.

All-cause, CVD (I00–I98), cancer (C00–C97), and other-cause (other causes than I00–I98 and C00–C97) mortality were analyzed. All-cause mortality is the sum of the three cause-specific groups.

### Independent variables

*Dental healthcare utilization* was assessed with the item ‘When was your latest visit to a dentist/dental hygienist?’ with the alternatives ‘Less than 1 year ago’, ‘1–2 years ago’, ‘3–5 years ago’, ‘More than 5 years ago’, and ‘I have never been to a dentist/dental hygienist’. The latter two alternatives were collapsed into a >5 years ago/never category in Tables 2–3 and Supplementary Table 1 due to the low prevalence (0.4%) of respondents in the ‘never’ category.

**Figure 2:**
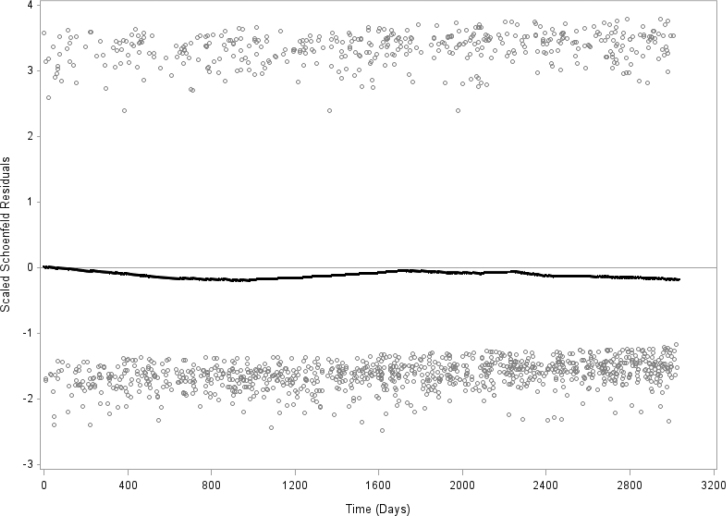
Schoenfeld residuals of frequency of dental healthcare (visit less than 1 year ago versus the other four groups) and all-cause mortality. Test of proportionality shows p = 0.265, which indicates proportionality. Men and women, N = 26,115. The Public Health Survey in Scania 2008.

Calculations in Tables 2, Figure 2, and Supplementary Table 1 were adjusted for *sex*. Calculations in Table 3 were matched for *sex*.

**Table 1:** Descriptive characteristics (%) of age, sex, socioeconomic status (SES), country of birth, chronic disease, leisure-time physical activity, daily smoking, alcohol consumption, and self-perceived dental health by frequency of dental healthcare.

	Frequency of dental healthcare					
	Less than 1 year ago	1–2 years ago	3–5 years ago	More than 5 years ago	Never	*p*-value
	*n* = 18,611	*n* = 4567	*n* = 1514	*n* = 1337	*n* = 86	
	68.4%	19.0%	6.7%	5.5%	0.4%	
**Age, yrs: mean ± SD^a^**	48.3 ± 16.7 (48.0–48.6)	40.5 ± 16.3 (40.0–41.1)	40.5 ± 15.9 (39.6–41.4)	47.0 ± 16.4 (45.8–48.1)	47.4 ± 18.7 (42.4–52.4)	< 0.001
**Sex^b^**						
Male	48.6 (47.8–49.5)	48.0 (46.2–49.9)	56.8 (53.8–59.9)	62.2 (59.0–65.4)	77.4 (66.3–88.6)	< 0.001
Female	51.4 (50.5–52.2)	52.0 (50.1–53.8)	43.2 (40.1–46.2)	37.8 (34.6–41.0)	22.6 (11.4–33.7)	
**Socioeconomic status (SES)^b^**						< 0.001
Higher non-manual	9.0 (8.5–9.5)	9.0 (8.0–10.0)	7.6 (5.9–9.2)	6.0 (4.3–7.6)	2.9 (0.0–7.0)	
Medium non-manual	13.8 (13.2–14.4)	14.8 (13.6–16.0)	11.7 (9.8–13.7)	10.8 (8.5–13.0)	4.7 (0.0–12.2)	
Lower non-manual	7.8 (7.4–8.3)	8.7 (7.7–9.7)	5.5 (4.1–6.9)	7.8 (5.9–9.7)	0.5 (0.0–1.6)	
Skilled manual	10.0 (9.5–10.6)	11.0 (9.9–12.2)	13.4 (11.1–15.7)	10.8 (8.7–12.8)	11.3 (2.2–20.4)	
Unskilled manual	11.7 (11.1–12.4)	13.4 (12.2–14.6)	14.3 (12.1–16.4)	16.8 (14.3–19.2)	21.8 (8.8–34.7)	
Self-employed/farmer	6.5 (6.1–7.0)	5.2 (4.4–6.0)	5.2 (3.8–6.6)	3.9 (2.4–5.4)	4.0 (0.0–10.1)	
Early retired	3.7 (3.3–4.0)	3.4 (2.7–4.1)	5.7 (4.1–7.2)	7.4 (5.7–9.1)	7.6 (1.3–13.8)	
Unemployed	3.3 (3.0–3.7)	4.8 (3.9–5.8)	7.4 (5.6–9.3)	6.4 (4.7–8.2)	14.8 (3.5–26.2)	
Student	7.4 (6.8–8.0)	11.8 (10.6–13.0)	10.8 (8.6–12.9)	4.0 (2.5–5.4)	7.6 (0.0–17.2)	
Old age pensioner	21.0 (20.4–21.7)	9.6 (8.7–10.5)	8.7 (7.1–10.2)	18.3 (15.9–20.6)	20.4 (10.3–30.5)	
Unclassified	4.7 (4.2–5.1)	6.8 (5.8–7.8)	7.8 (6.0–9.6)	5.9 (4.2–7.7)	4.5 (0.3–8.7)	
Long-term sick leave	0.9 (0.7–1.1)	1.4 (1.0–1.8)	2.0 (1.1–3.0)	2.0 (1.1–2.8)	-	
**Country of birth^b,c^**	15.3 (14.6–16.0)	21.2 (19.5–22.9)	29.4 (26.3–32.6)	26.5 (23.2–29.8)	59.3 (45.2–73.4)	< 0.001
**Chronic disease^b,c^**	28.4 (27.6–29.1)	26.7 (25.1–28.4)	30.6 (27.7–33.5)	36.9 (33.7–40.1)	24.3 (12.3–36.3)	< 0.001
**Low leisure-time physical activity^b^**	12.5 (11.8–13.1)	14.8 (13.4–16.2)	20.4 (17.9–22.9)	25.7 (22.7–28.6)	33.7 (19.2–48.2)	< 0.001
**Daily smoking^b^**	12.8 (12.1–13.4)	14.1 (12.7–15.5)	23.3 (20.5–26.0)	26.5 (23.6–29.5)	35.1 (20.9–49.3)	< 0.001
**Alcohol consumption past year^b^**						< 0.001
Never	10.5 (9.9–11.1)	13.4 (12.0–14.7)	17.2 (14.8–19.7)	20.7 (18.1–23.3)	39.1 (25.0–53.1)	
Once a month or more seldom	22.2 (21.5–23.0)	23.6 (22.1–25.1)	25.3 (22.6–28.0)	27.2 (24.3–30.1)	22.9 (9.1–36.7)	
2–4 times a month	35.5 (34.6–36.4)	38.7 (37.0–40.4)	33.4 (30.3–36.5)	28.2 (25.2–31.3)	16.0 (5.2–26.9)	
2–3 times a week	23.9 (23.2–24.7)	19.4 (18.0–20.9)	18.5 (16.0–21.1)	15.9 (13.6–18.3)	13.3 (1.3–25.2)	
At least 4 times a week	7.8 (7.4–8.3)	4.9 (4.1–5.7)	5.5 (4.2–6.8)	8.0 (6.1–9.8)	8.8 (0.5–17.0)	
**Self-perception of dental health**						< 0.001
Very good	32.1 (31.2–33.0)	23.8 (22.3–25.3)	9.7 (7.9–11.6)	9.8 (7.7–11.9)	24.0 (11.3–36.6)	
Rather good	47.8 (46.9–48.7)	47.7 (45.9–49.5)	36.6 (33.4–39.8)	25.9 (22.8–29.0)	24.8 (12.4–27.3)	
Neither good nor poor	13.5 (12.8–14.1)	18.5 (17.0–20.0)	31.0 (27.9–34.1)	26.6 (23.7–29.6)	26.1 (12.6–39.6)	
Rather poor	5.1 (4.7–5.5)	8.2 (7.2–9.3)	16.7 (14.2–19.1)	23.8 (20.9–26.6)	9.7 (1.7–17.6)	
Very poor	1.6 (1.3–1.8)	1.8 (1.3–2.3)	6.0 (4.3–7.6)	13.9 (11.6–16.2)	15.4 (5.1–25.7)	

The 2008–2016 Public Health Survey in Scania, Sweden. Total population *n* = 26,115. Weighted prevalence.

a *P*-value: Independent samples ANOVA-test, 2-tailed;

b *P*-value: Pearson Chi-Square test, 2-sided;

c The percentage depicts born abroad (as opposed to born in Sweden). The percentage depicts answer ‘yes’ to chronic disease as opposed to ‘no’. The values in parentheses are 95% confidence intervals for the mean or percent based on the bootstrap method with 1000 replicates.

**Table 2:** Hazard ratios (HRs) with 95% confidence intervals (95% CIs) of all-cause, cardiovascular (CVD), cancer, and other-cause mortality according to frequency of dental healthcare (dental healthcare utilization).

Cause of death	Model 0		Model 1		Model 2		Model 3		Model 4		Cases, No./ Persons at risk, no.
	HR	95% CI	HR	95% CI	HR	95% CI	HR	95% CI	HR	95% CI	
**All causes**											
Less than 1 year ago	1.0		1.0		1.0		1.0		1.0		986/18,611
1–2 years ago	0.8**	0.6–0.9	1.5***	1.2–1.8	1.4***	1.2–1.7	1.3*	1.0–1.6	1.3*	1.0–1.5	174/4567
3–5 years ago	**1.1**	0.9–1.5	2.4***	1.8–3.2	2.2***	1.7–3.0	1.7***	1.3–2.3	1.7***	1.3–2.2	94/1514
More than 5 years ago/never	2.1***	1.7–2.5	2.4***	1.8–2.8	1.9***	1.6–2.4	1.5***	1.2–1.9	1.4**	1.1–1.8	164/1423
**Cardiovascular disease**											
Less than 1 year ago	1.0		1.0		1.0		1.0		1.0		300/18,611
1–2 years ago	0.7	0.5–1.0	1.4	1.0–2.1	1.4	0.9–2.0	1.2	0.8–1.7	1.1	0.8–1.7	50/4567
3–5 years ago	1.3	0.9–2.0	2.9***	1.9–4.4	2.7***	1.8–4.1	1.9**	1.2–3.0	1.8*	1.1–2.8	36/1514
More than 5 years ago/never	1.8**	1.2–2.6	1.8**	1.3–3.0	1.5*	1.0–2.4	1.1	0.7–1.7	1.0	0.6–1.5	47/1423
**Cancer**											
Less than 1 year ago	1.0		1.0		1.0		1.0		1.0		395/18,611
1–2 years ago	0.6***	0.4–0.8	1.1	0.8–1.5	1.1	0.8–1.5	1.0	0.7–1.4	1.0	0.7–1.5	65/4567
3–5 years ago	0.9	0.6–1.5	1.9**	1.2–3.0	1.8*	1.1–2.9	1.6	1.0–2.6	1.6	1.0–2.6	31/1514
More than 5 years ago/never	1.7**	1.2–2.4	1.9***	1.3–2.7	1.8**	1.2–2.5	1.5*	1.0–2.1	1.5*	1.0–2.3	59/1423
**Others**											435
Less than 1 year ago	1.0		1.0		1.0		1.0		1.0		291/18,611
1–2 years ago	1.1	0.8–1.5	2.0***	1.5–2.8	1.9***	1.4–2.7	1.7**	1.2–2.4	1.7**	1.2–2.3	59/4567
3–5 years ago	1.3	0.7–2.3	2.7***	1.5–4.8	2.4**	1.3–4.2	1.8*	1.0–3.1	1.7	0.9–3.0	27/1514
More than 5 years ago/never	2.9***	2.0–4.1	3.2***	2.2–4.5	2.6***	1.8–3.8	2.0***	1.4–2.9	1.8***	1.3–2.7	58/1423

The 2008–2016 Scania public health survey with 8.3 years of follow-up. Men and women combined. Total population *n* = 26,115. Weighted.

Model 0 unadjusted. Model 1 adjusted for sex and age. Model 2 additionally adjusted for socioeconomic status (SES), country of birth (born in Sweden vs abroad), and chronic disease (no/yes). Model 3 additionally adjusted for smoking, leisure-time physical activity, and alcohol consumption. Model 4 additionally adjusted for self-perceived dental health.

Significance levels:

* *p* < 0.05,

** *p* < 0.01,

*** *p* < 0.001; Weighted Hazard Ratios. Bootstrap method (1000 replicates) for variation estimation.

**Table 3:** Odds ratios and 95% confidence intervals of all-cause, cardiovascular, cancer, and other-cause mortality according to the frequency of dental healthcare (dental healthcare utilization).

Cause of death	Model 1		Model 2		Model 3		Model 4		Number of	
	OR	95% CI	OR	95% CI	OR	95% CI	OR	95% CI	Case	Control
**All causes**									**1081**	**3243**
Less than 1 year ago	1.0		1.0		1.0		1.0		753	2667
1–2 years ago	1.5***	1.2–1.9	1.5***	1.2–1.9	1.3*	1.1–1.7	1.3*	1.0–1.7	133	326
3–5 years ago	2.3***	1.7–3.1	2.0***	1.4–2.8	1.6**	1.1–2.3	1.5*	1.0–2.2	69	109
More than 5 years ago/never	3.2***	2.5–4.1	2.5***	1.9–3.3	2.1***	1.6–2.8	1.9***	1.4–2.6	126	141
**CVD**									**382**	**1146**
Less than 1 year ago	1.0		1.0		1.0		1.0		269	927
1–2 years ago	1.3	0.9–1.9	1.2	0.8–1.9	1.1	0.7–1.7	1.1	0.7–1.6	42	114
3–5 years ago	2.7***	1.6–4.5	2.5**	1.5–4.4	1.8*	1.0–3.3	1.7	0.9–3.1	29	38
More than 5 years ago/never	2.2***	1.4–3.3	1.7*	1.1–2.6	1.3	0.8–2.1	1.2	0.7–1.9	42	67
**Cancer**									**501**	**1503**
Less than 1 year ago	1.0		1.0		1.0		1.0		362	1206
1–2 years ago	1.2	0.9–1.7	1.2	0.9–1.7	1.2	0.8–1.6	1.1	0.8–1.6	58	164
3–5 years ago	1.6*	1.0–2.6	1.6	1.0–2.6	1.5	0.9–2.4	1.3	0.8–2.2	29	60
More than 5 years ago/never	2.3***	1.6–3.4	2.1***	1.4–3.0	1.8**	1.2–2.7	1.7*	1.1–2.7	52	73
**Others**									**377**	**1131**
Less than 1 year ago	1.0		1.0		1.0		1.0		254	917
1–2 years ago	1.6*	1.1–2.3	1.7**	1.2–2.5	1.5	1.0–2.2	1.4	0.9–2.1	52	120
3–5 years ago	1.7	1.0–2.9	1.4	0.8–2.5	1.0	0.5–1.8	0.9	0.5–1.6	21	43
More than 5 years ago/never	3.7***	2.4–5.7	2.8***	1.8–4.5	2.3**	1.4–3.8	2.1**	1.3–3.7	50	51

Conditional logistic regression analyses in a matched case–control study nested within the prospective cohort 2008–2016 with 8.3-year follow-up, with the 2008 Scania public health survey as baseline. Men and women combined. *n* = 4324. Matched case–control study nested within the cohort. Cases and controls were matched 1:3 based on sex, age (±1 year), and baseline survey date; controls had equal or longer follow‑up. Odds ratios (ORs) and 95% confidence intervals (CIs) were estimated using conditional logistic regression. Model 1 matched for sex, age, and baseline survey date. Model 2 additionally adjusted for socioeconomic status, country of birth (born in Sweden vs born abroad), and chronic disease (no/yes). Model 3 additionally adjusted for smoking, leisure-time physical activity and alcohol consumption. Model 4 additionally adjusted for self-perceived dental health.

Significance levels:

* *p* < 0.05,

** *p* < 0.01,

*** *p* < 0.001.

*Age* was a continuous variable.

*Country of birth* was born in Sweden or born abroad.

*Socioeconomic status (SES)* (by occupation and relation to labor market) was defined in the categories nonmanual employees in higher, medium, and lower positions, skilled and unskilled manual workers, and self-employed/farmers. Categories outside the workforce additionally consist of the unemployed (in the labor force but seeking a job), early retired prior to age 65, old-age pensioners 65 and above, students, long-term sick leave, and unclassified.

*Chronic disease* was assessed with the item ‘Do you have any long-term disease, ailment or injury, any disability or other weakness?’, with the alternatives ‘Yes’ and ‘No’.

*Daily smoking* was assessed with the question ‘Do you smoke?’ with the alternatives daily smoker, non-daily smoker, and non-smoker. The latter two alternative answers were collapsed.

*Leisure-time physical activity* (LTPA) included ‘regular exercise (at least three times per week at least 30 minutes/occasion, leading to sweating), moderate regular exercise (exercising once or twice per week at least 30 minutes/occasion, leading to sweating), moderate exercise (more than 2 hours walking, cycling or equivalent activity/week), and low LTPA (less than 2 hours walking, cycling or equivalent activity/week)’ [25]. The LTPA item was dichotomized by collapsing the first three alternatives (which was used as reference in the multiple Cox regression analyses and nested case–control analyses) versus the low LTPA alternative. Low LTPA represents the lowest recommended level of physical activity.

*Alcohol consumption during the past year* was defined by the item ‘How often have you consumed alcohol during the past 12 months?’, with the alternative answers ‘daily or almost daily’, ‘several occasions per week’, ‘once per week’, ‘2–3 times per month’, ‘once per month’, ‘once or a few times per half year’, or ‘more seldom or never’.

*Self-perception of dental health* was assessed with the item ‘How do you perceive your dental health?’ with the alternatives ‘Very good’, ‘Rather good’, ‘Neither good nor poor’, ‘Rather poor’, and ‘Very poor’.

### Statistics

Prevalence (%) of all variables stratified by the five alternative answers to the dental healthcare utilization item was calculated. The differences between these five dental healthcare groups were assessed with a *t*-test for continuous variables and a chi-square test for categorical variables (*p*-values) (Table 1). Hazard ratios (HRs) with 95% confidence intervals (95% CIs) of all-cause, CVD, cancer, and other-cause mortality for the five dental healthcare groups were calculated in multiple survival (Cox) regression models. Five models were calculated: model 0 was unadjusted; model 1 adjusted for sex and age; model 2 additionally adjusted for SES; country of birth, and chronic disease; model 3 additionally adjusted for daily smoking, LTPA, and alcohol consumption; and model 4 additionally adjusted for self-perceived dental health (Table 2). Survival analyses restricted to the 40–80 years age interval with the same models were also conducted (Supplementary Table 1). Conditional logistic regression analyses with odds ratios (ORs) and 95% CIs analyzing models 1–4 with a nested case–control study design were conducted based on the present population cohort with each case (death within the 2008–2016 period) matched for age, sex, and baseline survey date with three controls within the cohort in model 1, model 2 additionally adjusted for SES, country of birth, and chronic disease, model 3 additionally adjusted for daily smoking, LTPA, and alcohol consumption, and model 4 additionally adjusted for self-perceived dental health (Table 3). The covariates included may be regarded as confounders because they are associated with the outcome mortality, while they are also associated with the exposure, without being in the pathway between exposure and outcome. Still, the risk of residual confounding remains a limitation. Follow-up days were followed from baseline to death or the last follow-up date (31 December 2016). Sampling variability may be analyzed without distributional assumptions of the study population by bootstrap analysis [26]. Accurate variance estimation on weighted data with CIs and *p*-values was calculated with bootstrap analyses, including 1000 replicates. An interaction term with time and dental healthcare was introduced to test the assumption of proportional hazards for all-cause mortality. Schoenfeld residuals were calculated for dental healthcare utilization and all-cause mortality (Figure 2). The SAS software version 9.4 was used in all analyses.

## Results

This study includes 26,115 respondents, 11,812 men and 14,303 women, after exclusion of 1947 respondents with internally missing values on any or several of the items included in the multiple analyses. Additionally, 136 of the baseline respondents were lost to follow-up. Table 1 shows that 68.4% of respondents had visited dental healthcare (dentist/dental hygienist) less than a year ago; 19.0%, 1–2 years ago; 6.7%, 3–5 years ago; 5.5%, more than 5 years ago; and 0.4%, never. The mean age of the groups who had visited dental healthcare 1–2 and 3–5 years ago was significantly lower than in the other dental healthcare groups. The groups who had visited dental healthcare 3–5 years ago, more than 5 years ago, and never were significantly overrepresented among men and underrepresented among women. Students had significantly more often visited dental healthcare 1–2 and 3–5 years ago, and significantly less often more than 5 years ago, compared to the dental healthcare less than 1 year ago group. The proportions of respondents born abroad were significantly higher in all four respondent groups that had visited dental healthcare more than 1 year ago than in the less than 1 year ago group. The respondent group that had visited dental healthcare more than 5 years ago reported chronic disease at baseline to a significantly greater extent compared to the groups that had visited dental healthcare less than 1 year ago, 1–2 years ago, and 3–5 years ago. Differences according to health-related behaviors were also observed. In the group that visited healthcare less than a year ago, 32.1% of respondents reported very good and 47.8% reported rather good dental health, that is, four of five respondents in this group reported very good or rather good health. Only 5.1% in this group reported rather poor and 1.6% very poor dental health. In contrast, in the group that visited dental healthcare more than 5 years ago, 9.8% reported very good dental health and 25.9% rather good health. A 23.8% proportion of this group reported rather poor dental health, and 13.9% very poor dental health.

Table 2 shows that all-cause mortality was significantly higher in respondent groups that visited dental healthcare 1–2 years ago, 3–5 years ago, and more than 5 years ago than in the reference group that visited dental healthcare less than 1 year ago throughout models 1–4. In the final model 4, the group that visited dental healthcare 1–2 years ago displayed an HR of 1.3 (95% CI 1.0–1.5), the group that visited dental healthcare 3–5 years ago, HR 1.7 (95% CI 1.3–2.2), and the group that visited dental healthcare more than 5 years ago**/**never, HR 1.4 (95% CI 1.1–1.8) of all-cause mortality compared to the reference group. The group that visited dental healthcare 3–5 years ago displayed significantly higher CVD mortality throughout the analyses, HR 1.8 (95% CI 1.1–2.8) in model 4. The group that visited dental healthcare more than 5 years ago**/**never had significantly higher cancer mortality throughout models 1–4, HR 1.5 (95% CI 1.0–2.3) in model 4. The groups that visited dental healthcare 1–2 years ago and more than 5 years ago both displayed significantly higher HRs of other cause mortality throughout models 1–4, HR 1.7 (95% CI 1.2–2.3) in model 1 and HR 1.8 (95% CI 1.3–2.7) in model 4, respectively.

The results of the conditional logistic regression analyses with a nested case–control study design, displayed in Table 3, show a more pronounced dose-response relationship between healthcare utilization and all-cause mortality than in Table 2. The 1–2 years ago group had a significant OR 1.3 (95% CI 1.0–1.7), the 3–5 years ago group a significant OR 1.5 (95% CI 1.0–2.2), and the >5 years ago**/**never group OR 1.9 (95% CI 1.4–2.6) compared to the <1 year ago reference group in the final model 4 and significantly higher ORs also in models 0–3. In the nested case–control analyses, the >5 years ago**/**never group also had OR 1.7 (95% CI 1.1–2.7) for cancer mortality and OR 2.1 (95% CI 1.3–3.7) for other-cause mortality, and significantly higher ORs in models 0–3.

Schoenfeld residuals for dental healthcare (visited dental healthcare less than 1 year ago versus the aggregate of the other four dental healthcare groups) and all-cause mortality show stability over 8.3 years (Figure 2). The interaction term between dental healthcare (visited dental healthcare less than 1 year ago versus the aggregate of the other four dental healthcare groups) and mortality across the 8.3-year period is not significant, *p* = 0.265, which indicates proportionality.

The supplementary Cox regression (survival) analyses restricted to the age strata 40–80 years in Supplementary Table 1 confirm the main results regarding patterns of all-cause mortality. Supplementary Table 1 also suggests significant associations between dental healthcare utilization and CVD, cancer, and other-cause mortality.

## Discussion

### Summary of key results

A 68.4% proportion had visited dental healthcare less than 1 year ago, 19.0% 1–2 years ago, 6.7% 3–5 years ago, 5.5% more than 5 years ago, and 0.4% never. The groups that had visited dental healthcare more than 1 year ago displayed increased HRs of all-cause mortality throughout the multiple survival analyses compared to the less than 1-year reference group. The group that had visited dental healthcare 3–5 years ago displayed significantly higher CVD mortality. The group that had visited dental healthcare more than 5 years ago**/**never had significantly higher cancer mortality. The groups that had visited dental healthcare 1–2 and more than 5 years ago displayed significantly higher other-cause mortality. The nested case–control study showed a clearer dose–response relationship between dental healthcare utilization and all-cause mortality, with the highest OR in the >5 year**/**never group, and significantly higher ORs of cancer and other-cause mortality in the >5 year**/**never group. The results show that dental healthcare utilization is associated with mortality. Most importantly, the results also highlight the importance of improving dental and oral healthcare, particularly among the elderly, to improve survival. The supplementary analyses restricted to the age strata 40–81 years in Supplementary Table 1 confirm the main results.

### Application and importance of the findings to the wider international community

The positive association between poor oral health and high mortality may, according to some authors, be explained by the fact that oral health problems and CVDs, to an important extent, have major risk factors in common, for example, age, low SES, and tobacco smoking [27]. We thus adjusted for sex, age, SES, country of birth, tobacco smoking, LTPA, and alcohol consumption in the multiple regression analyses to remove confounding and other sources of covariation. These variables may be regarded as confounders because they are associated with the outcome mortality, while they are also associated with the exposure, without being in the pathway between exposure and outcome. We have also adjusted for self-perceived chronic disease and self-reported dental health problems in the baseline survey questionnaire in 2008 to adjust for self-perceived systemic and dental health at baseline. They are also associated with both healthcare utilization and mortality.

The results support the notion that causal connections between frequency of dental healthcare and mortality may exist, although the risk of residual confounding in this observational study calls for caution. Plausible causal connections with CVD mortality have been outlined, including chronic inflammation as a contributor to the pathway of atherosclerosis [7, 8, 11]. Several plausible connections with other-cause mortality have also been outlined. The bacteria of dental plaque can spread in the connective tissue of the gingiva and cause infections, and even reach the blood, resulting in bacteremia [6]. Other plausible connections with other-cause mortality include tooth loss, which causes poor nutrition and poor eating behaviors. Problems with swallowing may lead to aspiration pneumonia in the elderly and frail [14]. Connections with cancer mortality include decreased intake of certain nutrients [28] that may cause gastrointestinal cancer [12].

Approximately 30% of those over 65 years of age had not been to a dental examination during the last 2 years [29], and a Swedish study also showed that adults with severe periodontitis that had refrained from dental care more than others during the last 3 years were also less able to handle the costs of routine care [30]. That a large proportion of the adult Swedish population do not visit the dentist regularly, paired with indications that those with the most needs refrain more than others, shows the need for measures to improve dental healthcare utilization. The results of this study are important as they add more to the need for reforms of the dental healthcare system in Sweden that may result in increased survival, which may also have a decreasing impact on the health gap between SES groups [31, 32]. The notion that oral and dental health should be treated differently from the general healthcare financial support may prove untenable. The reform subsidizing 90% of dental treatments for the elderly 67 years and above can only be evaluated prospectively in terms of the association between healthcare utilization and mortality in the future.

### Strengths and limitations

This study is large, population-based, and longitudinal. The participant population in the 2008 baseline public health survey has sufficient representativeness in relation to the general 18–80 years population in Scania in 2008. The risk of selection bias has been judged to be low to moderate (‘comparatively low’) based on a comparison of basic sociodemographic characteristics such as age, sex, and education between the respondent and parameter population in 2008 [33]. However, important selection bias, for example, higher propensity among people with high SES based on occupation or high income to respond and thus partly also distort associations, cannot be completely ruled out. Studies indicate that self-reported items provide valid information regarding key features of dental and oral health [34, 35]. The survival analyses with a nested case–control study design strengthen methodological robustness. Mortality data originate from the Swedish cause of death register, which has been used in most internationally published Swedish register-based studies, including mortality. The effects of dental healthcare utilization appear to be similar across age strata. We consequently included the younger age strata, which include very few deaths, but supplementary analyses including the 40–80 years strata were also conducted (Supplementary Table 1). Incidence calculations show that all-cause mortality in the 18–44 stratum was 86.1/100,000 among men compared to 81.6/100,000 in Sweden in 2012. All-cause mortality among women was 45.7/100,000 for women in the same age strata compared to 46.1/100,000 in Sweden. In the 45–64 age stratum, all-cause mortality among men was 573.9/100,000 compared to 464.0/100,000 in Sweden, and for women, 304.2/100,000 in the study compared to 303.1/100,000 in Sweden. Finally, in the age stratum 65–80, all-cause mortality was 3054.7/100,000 for men compared to 3060/100,000 in Sweden, and for women, 1769.7/100,000 compared to 2155/100,000 in Sweden [36, 37]. The item chronic disease was included to adjust for general health problems at baseline. Self-reported dental health has been included in the public health surveys in Scania every fourth year and in the national public health survey. The broadness of the chronic disease variable with its yes/no alternatives may be regarded as a weakness. Relevant confounders and covariates were included in the multiple analyses. However, there is still a risk of residual confounding caused by the absence in the survey of relevant factors such as income level or healthcare awareness. Some sources of bias, such as some recall bias, may remain. Direct validation of oral health and dental healthcare utilization was not conducted in connection with the present survey, but the dental healthcare utilization variable has been used in previous and later surveys in Scania as well as in the national survey in Sweden.

### Conclusion

A 68.4% proportion had visited dental healthcare less than 1 year ago; 19.0%, 1–2 years ago; 6.7%, 3–5 years ago; 5.5%, more than 5 years ago; and 0.4%, never. The groups that had visited dental healthcare 1–2 years ago, 3–5 years ago, and more than 5 years ago**/**never displayed increased HRs of all-cause mortality throughout the multiple survival analyses compared to the less than 1-year reference group. The results suggest that the frequency of visits to dental healthcare is associated with mortality independently of well-known confounders such as age, SES, and smoking. Reforms that facilitate access to dental healthcare may result in increased survival. However, given the risk of possible selection bias and residual confounding, especially regarding health data, results need to be interpreted with caution. Studies using robust registry data regarding dentist visits and health status are warranted.

## Supplementary Material


