# Supplementary material for: Dental healthcare utilization and mortality: a population-based prospective cohort study in southern Sweden
Source: Acta Odontol Scand. 2026 May 29;85:46190. doi: 10.2340/aos.v85.46190 (PMC13224782; doi:10.2340/aos.v85.46190)
Supplement: Supplementary file 1 [file AOS-85-46190-s1.pdf]

Supplementary material has been published as submitted. It has not been copyedited or typeset by Acta Odontologica Scandinavica.

Supplementary Table S1. Hazard ratios (HRs) with 95% confidence intervals (95% CIs) of all-cause, cardiovascular (CVD), cancer and other causes mortality according to frequency of dental healthcare (dental healthcare utilization) in the age strata 40-80 years. The 2008 -2016 Scania public health survey with **8.3** years follow-up. Men and women combined. Total population **n=18 208**. **Weighted.**

	Model 0		Model 1		Model 2		Model 3		Model 4		
**Cause of death**	**HR**	**(95%CI)**	**HR**	**(95%CI)**	**HR**	**(95%CI)**	**HR**	**(95%CI)**	**HR**	**(95%CI)**	**Cases, No./ Persons at risk, No.**
**All causes**											
Less than 1 year ago	1.00		1.00		1.00		1.00		1.00		969/13921
1-2 years ago	1.05	(0.86-1.28)	**1.45*** **	(1.19-1.77)	**1.37** **	(1.12-1.67)	**1.25***	(1.02-1.53)	**1.24***	(1.01-1.52)	166/2501
3-5 years ago	**1.69*** **	(1.25-2.28)	**2.56*** **	(1.92-3.41)	**2.24*** **	(1.69-2.96)	**1.79** **	(1.35-2.38)	**1.72*** **	(1.29-2.30)	94/812
More than 5 years ago/never	**2.27*** **	(1.85-2.79)	**2.23*** **	(1.79-2.78)	**1.88*** **	(1.51-2.35)	**1.48*** **	(1.19-1.86)	**1.41** **	(1.12-1.77)	161/974
**Cardiovascular disease**											
Less than 1 year ago	1.00		1.00		1.00		1.00		1.00		300/13921
1-2 years ago	0.94	(0.64-1.38)	1.34	(0.92-1.97)	1.23	(0.83-1.84)	1.14	(0.76-1.70)	1.12	(0.74-1.69)	49/2501
3-5 years ago	**1.90** **	(1.24-2.92)	**2.92*** **	(1.91-4.47)	**2.50*** **	(1.64-3.80)	**1.89** **	(1.22-2.92)	**1.75***	(1.11-2.75)	36/812
More than 5 years ago/never	**1.95** **	(1.31-2.92)	**1.83** **	(1.21-2.77)	1.48	(0.97-2.27)	1.09	(0.69-1.70)	0.98	(0.63-1.52)	47/974
**Cancer**											
Less than 1 year ago	1.00		1.00		1.00		1.00		1.00		387/13921
1-2 years ago	0.80	(0.57-1.13)	1.09	(0.78-1.53)	1.07	(0.75-1.51)	1.03	(0.73-1.46)	1.05	(0.74-1.48)	61/2501
3-5 years ago	1.36	(0.84-2.21)	**2.05** **	(1.27-3.29)	**1.91** **	(1.19-3.08)	1.66	(1.00-2.76)	**1.70***	(1.02-2.84)	31/812
More than 5 years ago/never	**1.79** **	(1.24-2.58)	**1.83** **	(1.26-2.65)	**1.66***	(1.14-2.42)	1.44	(0.99-2.10)	1.47	(0.96-2.25)	56/974
**Others**											
Less than 1 year ago	1.00		1.00		1.00		1.00		1.00		282/13921
1-2 years ago	**1.51***	(1.07-2.12)	**2.07*** **	(1.48-2.91)	**1.92*** **	(1.37-2.69)	**1.67** **	(1.19-2.36)	**1.62** **	(1.14-2.31)	56/2501
3-5 years ago	**1.90***	(1.03-3.51)	**2.85*** **	(1.57-5.18)	**2.39** **	(1.32-4.33)	**1.83***	(1.01-3.31)	1.71	(0.93-3.15)	27/812
More than 5 years ago/never	**3.31*** **	(2.34-4.69)	**3.27*** **	(2.29-4.68)	**2.63*** **	(1.84-3.76)	**1.99*** **	(1.38-2.89)	**1.86*** **	(1.30-2.67)	58/974

Model 0 unadjusted. Model 1 adjusted for sex and age. Model 2 additionally adjusted for socioeconomic status (SES), country of birth (Sweden vs abroad) and chronic disease (no/yes). Model 3 additionally adjusted for smoking, leisure-time physical activity and alcohol consumption. Model 4 additionally adjusted for self-perception of dental health.
Significance levels: * p<0.05, ** p<0.01, *** p<0.001; Weighted Hazard Ratios. Bootstrap method (1000 replicates) for variation estimation.
